# Squamous cell carcinoma on top of urethral stricture: case report and review of the literature

**DOI:** 10.3332/ecancer.2013.304

**Published:** 2013-04-11

**Authors:** Ahmed Fouad Kotb, Doaa Attia, Asmaa Mohamed Ismail, Ahmed Elabbady

**Affiliations:** Urology department, Faculty of Medicine, Alexandria University, Al Khartom Square, Azarita, Alexandria, Egypt.

**Keywords:** urethral stricture, penile cancer, pseudoepitheliomatous hyperplasia

## Abstract

**Introduction:**

Urethral stricture is a common urological condition, resulting from trauma or venereal infections. The aim of our study was to report a rare case of squamous cell carcinoma of the penis and pseudoepitheliomatous hyperplasia (PEH) of scrotal skin, on top of repeatedly managed urethral stricture which was of unknown aetiology.

**Methods:**

A Medline search of publications studying the association of urethral stricture with penile cancer was done.

**Results:**

Two case reports were identified that described two occurrences, which were separated by a few months.

**Conclusion:**

Repeated management of urethral stricture with visual urethrotomy or urethral dilation may result in a chronic inflammatory status, predisposing to PEH and squamous cell carcinoma of the genital organs.

## Introduction

Urethral stricture is a urological condition of high frequency in Egypt and many other developing countries [1]. Its association with penile cancer is not well defined in the literature. Penile carcinoma is an uncommon condition and represents less than one per cent of all male cancers; its incidence increases most probably in uncircumcised men and those with multiple sexual partners.

Few studies in the literature have reported the association of urethral stricture and its management with the development of penile cancer. The aim of our work was to report an interesting case report, managed by our department, and to provide a concise review of similar cases in the literature.

## Case report

We encountered a 55-year-old man, complaining of difficulty in micturition and tenderness in the ventral aspect of the penis. The patient gave a history of urethral stricture that was diagnosed five years ago. According to the patient, the stricture was in the anterior part of the urethra, for which he was treated repeatedly with endoscopic visual urethrotomy, followed by multiple urethral dilations either with metal urethral dilators or sometimes with Nelaton urethral catheters.

Six months ago, his attending urologist decided to perform a urethral exteriorisation on the patient. The patient had his symptoms partially improved, but later on he began to complain of a recurrence, and so decided to visit our department. The patient is diabetic and did not provide any history of urethral trauma or sexually transmitted diseases.

When presenting to us, the scrotal wall was oedematous and unhealthy. The penis was exteriorised and highly suspicious for malignancy, especially with the hardness appreciated in the glans penis. A suprapubic catheter was inserted, and antibiotics were initiated with strict control of his Diabetes Mellitus (DM). A wedge biopsy was taken from the urethra and surrounding penile tissue. Inguinal lymph nodes were not palpable. A multiphasic CT done on the abdomen and pelvis was free of suspicious masses. The pathological analysis confirmed the diagnosis of squamous cell carcinoma. The patient was counselled and agreed to proceed for total penectomy and partial scrotectomy.

Final pathology reported moderately differentiated squamous cell carcinoma involving the urethra, penile skin and underlying subepithelial tissue, with a 4-cm free surgical margin. The scotal wall was reported to have PEH and free of malignancy.

The patient required a long hospital stay, exceeding 2 weeks, for medical treatment of the local infection of his wound and proper control of DM (Clavien grade II). The patient is currently a few months post-surgery, doing very well but, for psychological reasons, refusing to let us remove the suprapubic catheter.

## Methods

A medline review of all related publications in English was done. Search was done using the keyword urethral stricture, in association with squamous cell carcinoma, urethral cancer, and penile cancer.

## Results

Two case reports could be identified in the literature that were briefly discussed and correlated with our case report. The mean age of the patients was 65-years-old. The penile cancer was identified within few months, following the diagnosis of urethral stricture.

## Discussion

Reviewing the literature confirmed the rarity of the disease. We could only identify two case reports with urethral stricture that within a few months, turned out to be squamous cell carcinoma of the penis. Kathpalia *et al* [2] reported a case of 76-year-old man diagnosed with urethral stricture in the bulbar urethra and treated by urethral dilation. Four months later, the patient presented with ulcerative lesion on the penis and was treated by partial penectomy.The authors concluded that urethral tumours may arise from areas of urethral stricture, and human papilloma virus may play a role as a causative factor in both diseases. Malik *et al* [3] also reported a case of 55-year-old man, presenting with urethral stricture that was treated with visual urethrotomy. A penile mass then developed within few months but it was firstly diagnosed as periurethral abscess. When the swelling increased and the penis became fungated, penectomy was done confirming the diagnosis of penile cancer. Venereal diseases, especially herpetic infections, were suspected as an aetiological factor in both diseases. The first case was uncircumcised, and the second case had delayed circumcision at 13 years of age.

Our case is unique for many reasons; our patient who was circumcised during infancy did not have multiple sexual relationships during his life, is married, both he and his wife are clinically free of venereal disease, and had never previously complained of urethral discharge. He is a diabetic and a heavy smoker. Table 1 illustrates the data of the available case reports in relation to our case.

In contrast to the above case reports, the presentation of our case with penile cancer was late, occurring many years after the initial diagnosis of urethral stricture. Our patient was treated repeatedly with visual urethrotomy and urethral dilation. Does the recurrent irritation of the urethra with medical instruments, coupled with the badly controlled DM, result in a direct causative factor for the development of squamous cell carcinoma? We cannot confirm whether this is the truth or if the two diseases were just associations, but the long period between the two occurrences and the association with PEH of scrotal skin may make the urethral stricture and, specifically, its recurrent management a possible direct cause.

PEH is a benign condition, characterised by hyperplasia of the epidermis and closely resembling squamous cell carcinoma. It may be present in conditions characterised by prolonged inflammation and/or chronic infection [4]. Few case reports have been published showing the possible association of PEH and malignancies [5, 6]. To our knowledge, our case is the first in the literature to describe the association of squamous cell carcinoma of the penis with PEH of the scrotum. The two pathologies may be attributed to the longstanding infection and inflammatory response.

## Conclusion

Multiple interventions for the treatment of urethral stricture may result in a chronic inflammatory status, predisposing to PEH and squamous cell carcinoma of the urethra and genital organs.

## Figures and Tables

**Table 1.** Illustration of the available case reports

Parameters	Case report [2]	Case report [3]	Our case report
Age	76 years	55 years	55 years
Circumcision	Uncircumcised	Delayed circumcision	Infantile circumcision
Site of stricture	Bulbar urethral stricture	Bulbar urethral stricture	Penile and bulbar urethral strictures
Time to develop malignancy	4 months	3 months	5 years
Presentation	Ulcerative lesion at glans penis	Penile periurethral mass	Ventral penile masses involving glans and whole exteriorised urethra, with associated scrotal wall changes
Treatment	Partial penectomy	Total penectomy	Total penectomy
